# Lung function and exhaled nitric oxide in healthy unsedated African infants

**DOI:** 10.1111/resp.12579

**Published:** 2015-07-01

**Authors:** Diane Gray, Lauren Willemse, Ane Visagie, Emilee Smith, Dorottya Czövek, Peter D Sly, Zoltán Hantos, Graham L Hall, Heather J Zar

**Affiliations:** 1 Department of Paediatrics and Child Health, Red Cross War Memorial Children's HospitalCape Town, South Africa; 2 MRC Unit, Child and Adolescent Lung HealthCape Town, South Africa; 3 Division of Epidemiology and Biostatistics, School of Public Health and Family, University of Cape TownCape Town, South Africa; 4 Department of Medical Physics and Informatics, University of SzegedSzeged, Hungary; 5 Queensland Children's Medical Research Institute, University of QueenslandPerth, Western Australia, Australia; 6 Telethon Kids Institute, University of Western AustraliaPerth, Western Australia, Australia

**Keywords:** African, infants, lung function, reference data

## Abstract

**Background and objective:**

Population-appropriate lung function reference data are essential to accurately identify respiratory disease and measure response to interventions. There are currently no reference data in African infants. The aim was to describe normal lung function in healthy African infants.

**Methods:**

Lung function was performed on healthy South African infants enrolled in a birth cohort study, the Drakenstein child health study. Infants were excluded if they were born preterm or had a history of neonatal respiratory distress or prior respiratory tract infection. Measurements, made during natural sleep, included the forced oscillation technique, tidal breathing, exhaled nitric oxide and multiple breath washout measures.

**Results:**

Three hundred sixty-three infants were tested. Acceptable and repeatable measurements were obtained in 356 (98%) and 352 (97%) infants for tidal breathing analysis and exhaled nitric oxide outcomes, 345 (95%) infants for multiple breath washout and 293 of the 333 (88%) infants for the forced oscillation technique. Age, sex and weight-for-age z score were significantly associated with lung function measures.

**Conclusions:**

This study provides reference data for unsedated infant lung function in African infants and highlights the importance of using population-specific data.

**Summary at a Glance**

This is the first description of lung function in healthy African infants. Lung function in African infants differs to that of European infants. Population-specific reference data are important.

## Introduction

Measuring lung function in early life allows assessment of the determinants of early respiratory health and may provide a prognostic and susceptibility measure of respiratory disease. This is especially relevant to low- and middle-income settings (LMICs), where there is a very high burden of childhood respiratory disease and very limited data on infant lung function. Moreover, it is now well established that early lung function tracks through to adulthood, predicting diminished lung function and chronic respiratory disease in later life.^1^,^2^

Appropriate lung function data are essential to accurately assess the impact of early life factors on lung function and to distinguish between health and disease. Using reference data developed from differing equipment or methods or derived from different populations can lead to misinterpretation of the test results.^3^ Hence, developing reference ranges for tests and for a specific population is important. Non-invasive lung function measures undertaken in unsedated infants have been developed,^4^–^8^ furthering the use of infant lung function testing in epidemiological settings and the development of robust reference ranges. Reference data for Caucasian infants for both sedated^9^–^13^ and unsedated^14^,^15^ lung function tests have been published. Reference data for infant lung function in non-Caucasian subjects^16^ are limited and no data are available for African infants, nor for infants from LMICs.

This study aimed to describe the lung function in healthy South African infants living in low socioeconomic conditions typical of LMICs and to provide reference data for tidal breathing parameters, exhaled nitric oxide, multiple breath washout and forced oscillation technique measures.

## Methods

### Setting

Infant lung function testing was undertaken as part of a birth cohort study, the Drakenstein Child Health study (DCHS).^17^ This birth cohort study in a peri-urban, low socioeconomic community in South Africa aims to investigate the epidemiology and aetiology of childhood respiratory illness and the determinants of child lung health. The lung function testing site was established at the local public hospital.

### Participants

Healthy infants, enrolled in the DCHS, underwent testing at 5 to 11 weeks. Infants were excluded if they were born preterm (<37 weeks gestation) or had a history of respiratory distress at birth or prior respiratory tract infection. Gestation was assessed by antenatal ultrasound or by a combination of last menstrual period, antenatal examination of pubic symphysis fundal height and birth weight, when ultrasound was not available.

### Antenatal and early life data

Information regarding antenatal, birth and early life exposures and events were collected by questionnaire at scheduled antenatal and study visits.

### Lung function measurements

Lung function measurements included tidal breathing (TBFVL), exhaled nitric oxide (eNO), sulphur hexafluoride multiple breath washout (MBW) and the forced oscillation technique (FOT). Infants were tested from July 2012 to December 2013 for TBFVL, eNO and MBW and, for operational reasons, from October 2012 to December 2013 for FOT. Lung function measures were taken in unsedated infants during behaviourally assessed quiet sleep, as previously published.^18^,^19^ All testing conformed to American Thoracic Society/European Respiratory Society (ATS/ERS) guidelines.^20^–^22^

TBFVL and eNO measures were collected simultaneously using the Exhalyser D with ultrasonic flow meter and CLD 88 Exhalyzer chemoluminescent analyser (Ecomedics AG, Duernton, Switzerland) as previously described.^6^,^18^ A 90-s measurement epoch was made during quiet TBFVL. Recordings were included if >30 consecutive breaths (free of sighs, respiratory pauses, irregular volume breaths or air leak) of TBFVL were recorded and analysed according to international guidelines.^23^ Mean TBFVL measures, eNO and NO output were calculated using analysis software (Wbreath v3.28.0. Ndd Medizintechnik, AG, Zurich, Switzerland).

MBWs were performed using 4% SF6 as a tracer gas and ultrasonic flow meter (Spirison, Ecomedics, Duernten, Switzerland) with acquisition and analysis software (Wbreath v3.28.0, Ndd Medizintechnik AG) as reported previously.^5^,^18^,^24^ The washout period began after a 10 breath equilibrium period was obtained at the end of the tracer gas wash-in and continued until tracer gas eliminated from the lungs. The recordings were defined as acceptable for analysis if they were free of leak, occurred during quiet sleep, the wash-in equilibrium period had a stable tidal volume with inspiratory and expiratory end tidal inert gas concentration variability of <1%; no sighs, breath holds or irregular breathing pattern occurred within 10 breaths of the wash-in plateau or 10 breaths after the SF6 concentration had returned to baseline, 1/40th the concentration at start of washout.^22^ Three successful recordings were taken. Test repeatability was defined as functional residual capacity (FRC) means within 25% and lung clearance index (LCI) within 1 turnover of each other. The mean FRC, LCI and moment ratios of the three tests were reported. If only two successful tests were obtained, the tests were reported as the mean of two tests if the FRC mean was within 10% of the lower value and the LCI were within 1 turnover of each other. Mean dilution numbers, M0, M1 and M2 are calculated from the area under the curve of the end tidal inert gas concentration and lung turnovers measured.^22^ The first moment ratio was calculated as M1/M0 and the second as M2/M0, using the automated mode within the Wbreath software.

The FOT measurement was made with purpose built equipment. (University of Szeged, Hungary) as previously reported.^19^,^25^ A composite medium frequency signal (8–48 Hz) was delivered to the infants via a wave-tube through a facemask covering the mouth and nose. A minimum of five technically acceptable 30-s data epochs were collected. Recordings (or short segments of them) that contained breath holds, cries, irregular breathing or leaks were excluded. The epochs required at least 10 regular consecutive breaths to be included in analysis. The mean values of respiratory system impedance spectra were evaluated by fitting a resistance (R)—compliance (C)—inertance model to the measured data.^25^

### Ethics

The study was approved by the Faculty of Health Sciences, Human Research Ethics Committee, University of Cape Town (401/2009) and by the Western Cape Provincial Health Research Committee. Mothers gave informed, written consent in their first language for their infants to participate.

### Statistical analysis

Descriptive statistics were performed using STATA 13 (STATA Corporation, College Station, TX, USA). Data are presented as mean and standard deviation (SD) for normally distributed variables and median and 25–75% confidence intervals for non-normally distributed variables. Comparisons between populations are presented as sample means and standard error. The intra-subject coefficient of variation (CoV) was calculated as the ratio of each parameter SD over the parameters mean per study participant. For the TBFVL and eNO, this is the mean value of included breaths, and for MBW and FOT the mean of the results from each test.

Reference equations were fitted using a stepwise linear regression model with the significance level for removal set to > 0.05. The linear predictions from the reference equations were used to determine the predicted values for each lung function measurement. Details of statistical analysis are given in Supplementary Appendix S1.

## Results

**Figure 1:**
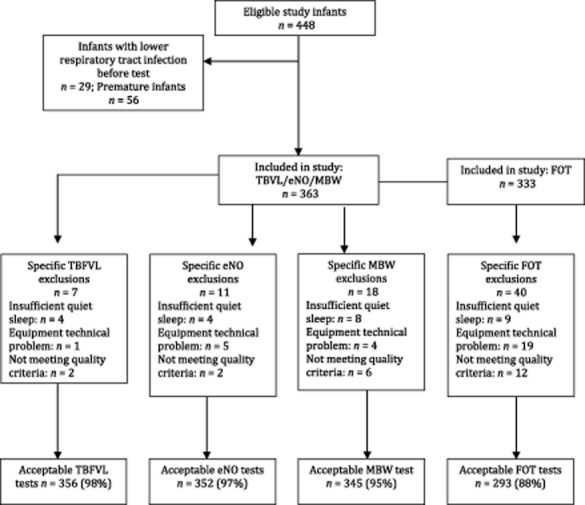
Cohort description before exclusions (*n* = 448).

Of the 448 infants, 85 infants were excluded (29 had had a prior lower respiratory tract infection and 56 were preterm), giving 363 infants eligible for inclusion (Fig. 1). The median age of infants was 7.4 weeks with an equal gender distribution (Table 1). Demographic and socioeconomic factors are shown in Table 2. The demographics by enrolment site are shown in Supplementary Table S1. Success rates for testing were 356 (98%) of tidal breathing tests, 352 (97%) eNO and 345 (95%) of MBW tests; and for the FOT, 293 of the 333 (88%). Reasons for unsuccessful testing are detailed in Figure 1.

**Table 1:** Anthropometric characteristics (*n* = 363)

	Median	25–75%	Range
Age (weeks)	7.4	6.6–8.1	5.3–11.4
Weight (kg)	4.8	4.4–5.4	2.8–6.9
Weight for age z score†	−0.32	−0.97–0.44	−4.2–2.1
Length (cm)	55	53–57	47–63
Height for age z score	−0.66	−1.7–0.2	−5.2–2.8
Gestational age (weeks)	39	38–40	37–43
Birth weight (kg)	3.1	2.8–3.5	1.8–4.3
Birth weight z score	−0.7	−1.4 to −0.1	−3.7–1.6
Birth length (cm)	51	48–53	35–58
Birth length z score	0.003	−0.8–0.9	−7.1–3.3

† Z scores calculated using the World Health Organization Child Growth Standards^26^ and updated Fenton newborn growth charts.^27^

**Table 2:** Demographics and socioeconomic characteristics of participants

	Total (*n* = 363)
	*n* (%)
Male sex	181 (50)
Maternal HIV infection	69 (19)
Maternal smoking in pregnancy	121 (34
Caesarean section	69 (19)
Exclusively breastfed	148 (41)
Maternal SES	
Lowest SES	97 (27)
Low-moderate SES	85 (23)
Moderate-high SES	94 (26)
High SES	87 (24)
Ethnicity	
African	176 (48)
Mixed ethnicity	187 (51)

HIV, human immunodeficiency virus; SES, socioeconomic status.

### TBFVL

The TBFVL group outcomes are listed in Table 3. The CoV ranged from 7.1% for minute ventilation to 20% for the ratio of time to peak tidal expiratory flow over total expiratory time (t_PTEF_/t_E_). The association with known and possible predictors of TBFVL parameters are shown in Supplementary Tables S2–S7. Weight-for-age z score was positively associated with tidal volume, minute ventilation and mean tidal flows; and negatively associated with respiratory rate. Weight was not a predictor of t_PTEF_/t_E_. Males had an increased tidal volume (2.3 mL higher compared with females; 95% confidence interval (CI) 1.2 to 3.3, *P* < 0.001) and lower t_PTEF_/t_E_ (2.8% lower compared with females; 95% CI −5.3 to −0.3, *P* = 0.03).

**Table 3:** Lung function values in healthy South African infants

	Mean (SD)	Range	Median (25–75%)	CoV med (25–75%)
Tidal breathing parameters *n* = 356				
Tidal volume mL	34.9 (6.3)	18.86–54.24	34.52 (30.78–39.0)	7.7 (6.3–9.9)
Respiratory rate n/min	48.1 (11.9)	24.8–104.10	46.4 (40.10–54.25)	8.0 (6.7–10.2)
Minute ventilation mL/min	1627.0 (307.6)	986.5–2836.0	1598 (1398–1810)	7.1 (5.9–9.0)
Mean inspiratory tidal flow mL/s	60.6 (10.6)	34.85–102.0	59.2 (53.4–66.5)	7.7 (6.2–10.3)
Mean expiratory tidal flow mL/s	50.0 (11.96)	25.00–92.93	48.3 (41.0–56.9)	9.1 (7.4–11.5)
t_PTEF_/t_E_ %	39.8 (12.1)	12.87–82.17	39.9 (31.6–46.7)	20.1 (16.8–25.2)
Exhaled nitric oxide *n* = 352				
eNO ppb	10.1 (6.8)	0.20–46.20	9.0 (5.0–13.7)	4.3 (3.4–6.5)
NO output nL/min	33.1 (21.3)	0.3–100.0	30.6 (16.5–47.5)	8.0 (6.6–10.2)
Multiple breath washout *n* = 345				
FRC mL	77.97 (17)	47.3–162.7	75.1 (66.1–86.6)	5.4 (3.3–7.8)
M0/M1	2.1 (0.1)	1.5–2.6	2.1 (2.0–2.2)	3.8 (2.2–5.5)
M0/M2	8.2 (1.1)	3.9–11.9	8.1 (7.4–8.9)	8.4 (4.9–12.4)
LCI	7.2 (0.4)	5.35–8.58	7.16 (6.91–7.46)	4.0 (2.4–5.8)
Forced oscillation technique *n* = 293				
R cmH_2_O/s/L	48.6 (15.7)	22.0–119.7	45.6 (38.1–57.2)	4.9 (3.0–8.0)
C mL/cmH_2_O	0.95 (0.44)	0.28–3.32	0.87 (0.68–1.15)	11.0 (6.1–17.9)
*f*res Hz	22.6 (6.1)	10.7–42.0	21.2 (18.7–25.4)	5.4 (3.3–9.6)

C, respiratory system compliance; CoV, intra-individual intra-test coefficient of variation; eNO, exhaled nitric oxide; FRC, functional residual capacity; *f*res, resonant frequency; LCI, lung clearance index; M0/M1, first mean dilution number; M0/M2, second mean dilution number; R, respiratory system resistance; t_PTEF_/t_E_, ratio of time to peak tidal expiratory flow over total expiratory time.

### eNO

Group results for measured eNO and NO output are detailed in Table 3. The median intra-subject CoV was small, 4.3% for eNO and 8.0 % for NO output. Associations are shown in Supplementary Tables S8 and S9. Age had a small but significant positive association with both eNO and NO output. Weight had a weak association with eNO but not with NO output. Gender was not associated with NO measures.

### Multiple breath washout

The group results for FRC, the first and second moment ratios (M0/M1 and M0/M2) and LCI are detailed in Table 3. The median intra-subject CoV was small for MBW measurements, FRC 5.4%, LCI 4.0%, M0/M1 3.8% and M0/M2 8.4%. The results of the multivariate analysis for associations of MBW measurements are shown in Supplementary Tables S10–S13. Age, weight-for-age z score and birth weight z score were associated with FRC. No size associations were found for the moment ratios or LCI. No predictive equation was appropriate for the moment ratios or LCI and the predicted upper and lower limits of normal were defined using the observed mean and SD.

### FOT

The FOT group outcomes are detailed in Table 3. The median intra-subject CoV was 4.9% for R, 11% for C and 5.4% for resonant frequency (*f*res). Associations are shown in Supplementary Tables S14–S16. Length was positively associated with R, C and *f*res. Weight was positively associated with R. Male gender was associated with increased R, decreased C and a slightly higher *f*res. Stratification by gender was considered but did not lead to meaningful differences in reference equations.

The South African reference equations calculated from these data are listed in Supplementary Table S17. The observed data were compared with the values predicted by these reference equations and the published European reference equations^15^ in Table 4. The European prediction equations did not satisfactorily predict the South African values.

**Table 4:** Comparison between measured and predicted values for South African and European models

	Observed values mean (standard error)	Predicted values mean (standard error)	Reference values^15^ mean (standard error)
Tidal volume mL	34.90 (0.34)	34.90 (0.21)	33.39 (0.19)*
Respiratory rate n/min	48.11 (0.63)	48.08 (0.20)	45.93 (0.31)*
Minute ventilation mL/min	1627.08 (16.33)	1624.67 (5.86)	†
Mean inspiratory tidal flow mL/s	60.55 (0.56)	60.50 (0.23)	57.44 (0.36)*
Mean expiratory tidal flow mL/s	50.05 (0.63)	49.96 (0.17)	†
t_PTEF_/t_E_ %	39.73 (0.63)	39.71 (0.15)	†
eNO ppb	10.11 (0.36)	10.14 (0.09)	13.13 (0.15)*
NO output nL/s	8.94 (0.24)	8.92 (0.05)	3.70 (0.03)*
FRC mL	78.01 (0.92)	77.95 (0.34)	107.18 (0.47)*
M0/M1	1.54 (0.01)	1.54 (0.00)	†
M0/M2	5.92 (0.04)	5.92 (0.01)	†
R cmH_2_O/s/L	48.55 (0.92)	48.78 (0.19)	‡
C mL/cmH_2_O	0.95 (0.03)	0.94 (0.01)	‡
*F*res Hz	22.64 (0.36)	22.65 (0.09)	‡

* Statistically significant difference with *P* < 0.001.

† No reference equations fitted

‡ Not tested.

## Discussion

This large cohort study provides reference data for lung function measures in healthy unsedated South African infants early in life, providing reference equations that can be used in clinical and epidemiological studies in African infants. This study provides novel data for infants living in an area with a high burden of childhood respiratory disease. In addition, this study provides the first reference data for a novel FOT in healthy 6-week-old infants, a safe and feasible measure of respiratory system impedance in unsedated infants.

Strengths of this study include the large size of the cohort, the collection of data using rigorous methodology by the same trained staff under similar conditions, which adhered to ATS/ERS guidelines; the strict quality control during data collection and analysis and the high success rate of testing. Factors contributing towards a high success rate were dedicated quiet testing space with a well-trained and experienced team skilled in coordinating infant sleep and testing; participant families being willing to wait at the testing site for infant sleep and the fact that repeat testing was attempted in failed cases.

A limitation of the study is the narrow age range in which lung function was measured, which limits generalizability of these reference data to older children. In addition, and similar to the previous published reference data,^15^ our model had low *R*^2^ values (Supplementary Tables S2–S16), suggesting that there are additional unaccounted factors influencing early lung function. However, this reference data may be useful in epidemiological studies and in longitudinally assessing the impact of early life exposures on lung health, which is currently being done as part of the DCHS. Children from LMICs are at particular risk of respiratory disease,^28^ hence understanding the impact of early exposures on lung growth and function in these settings is key.

### Comparison with data from high-income settings

There are few data on lung function parameters in healthy, unsedated infants using the same techniques. Similar TBFVL, eNO and MWB data have been published in European and Australian infants.^15^,^29^

The intra-test intra-individual CoV, a measure of test repeatability, of the TBFVL measures were similar to that previously reported using the same techniques in 6-week-old infants.^8^,^15^ Tidal volume, respiratory rate and flows were similar to those previously reported in sedated and unsedated infants, although South African infants had a slightly larger tidal volume and minute ventilation compared with European infants.^15^,^29^ However, South African infants were an average of 2 weeks older and 0.5 kg heavier than the European infants, accounting for this difference.^15^,^29^

The eNO was lower in South African compared with European infants, mean ± SD eNO of 10.1 ± 6.8 in South African versus 14.3 ± 6.0 European infants.^15^

South African infants had a lower FRC and higher LCI (poorer gas mixing efficiency) compared with European and American studies.^15^,^30^ Fuchs *et al*. reported measures using the same techniques in European infants of a similar age (mean ± SD 7.2 ± 0.4 weeks vs. 6.75 ± 0.6 weeks in European infants). South African infants had a significantly lower FRC compared with European infants (mean ± SD FRC 78 ± 17 mL vs. 102 ± 16 mL in European infants). These differences in MBW results may be accounted for in part by differing hardware and software, but may also represent different populations with particular exposures. These data suggest that infants living in low socioeconomic conditions with high exposure to air pollutants, such as tobacco smoke, have early evidence of impaired lung growth and function compared with infants from high-income areas.

No studies have been published of normative data in 6-week-old infants using this version of the FOT. However, the R and C CoV are similar to those collected using the FOT in newborn infants^25^ and older children.^31^ Direct comparisons cannot be made between R and C measured with FOT and other techniques, such as the interrupter technique (IT) and single breath occlusion technique (SOT). However R, which is relatively frequency independent, is similar to that measured with SOT.^11^,^32^ In contrast, C measured with FOT is lower than that measured with the SOT.^11^,^32^ R measured here was slightly higher than R measured in unsedated European infants using the IT.^15^ Differing measurement techniques may have contributed to the reduced impedance in South African infants, but may also be due to population differences.

This reduced flow ratio found in males is consistent with previous reports of lower lung function in male as compared with female infants.^33^–^36^ Male infants had a lower C, higher R and higher resonant frequency. This is similar to previous reports of lower C in healthy male compared with female infants.^11^

The previously published equations in Caucasian European infants did not fit the South African data well, despite the data being collected under similar conditions. This highlights the importance of reference data being specific not only for the equipment and method used, but also for the population studied.

In conclusion, this paper is the first description of healthy reference ranges of lung function data in African infants, and provides data for infants living in an area with a high burden of childhood respiratory disease. These data highlight the importance of using reference data that is specific to the population studied.

## Abbreviations

ATS/ETS: American Thoracic Society/European respiratory Society
C: compliance
CI: confidence interval
CoV: coefficient of variation
DCHS: Drakenstein Child Health study
eNO: exhaled nitric oxide
FOT: forced oscillation technique
FRC: functional residual capacity
*f*res: resonant frequency
IT: interrupter technique
LCI: lung clearance index
LMIC: low and middle income settings
MBW: multiple breath washout
R: resistance
SD: standard deviation
SOT: single breath occlusion technique
TBFVL: tidal breathing
t_PTEF_/t_E_: time to peak tidal expiratory flow over total expiratory time

## Supporting Information

Additional Supplementary Information can be accessed via the *html* version of this article at the publisher's website.

**Appendix S1** Statistical analysis. **Table S1** Demographics and socioeconomic characteristics of participants by study site. **Table S2** Univariate and multivariate analysis for tidal volume. **Table S3** Univariate and multivariate analysis for respiratory rate. **Table S4** Univariate and multivariate analysis for minute ventilation. **Table S5** Univariate and multivariate analysis for mean tidal inspiratory flow. **Table S6** Univariate and multivariate analysis for mean tidal expiratory flow. **Table S7** Univariate and multivariate analysis for time to peak tidal expiratory flow over total expiratory time (t_PTEF_/t_E_). **Table S8** Univariate analysis, multivariate analysis and reference equation for exhaled nitric oxide (eNO; A); (B) log(eNO). **Table S9** Univariate and multivariate analysis for nitric oxide output. **Table S10** Univariate and multivariate analysis for functional residual capacity (FRC). **Table S11** Univariate analysis for the first moment ratio (M0/M1). **Table S12** Univariate analysis for second moment ratio (M0/M2). **Table S13** Univariate analysis for the lung clearance index (LCI). **Table S14** Univariate and multivariate analysis for respiratory system resistance (R). **Table S15** Univariate and multivariate analysis for respiratory system compliance (C). **Table S16** Univariate and multivariate analysis for resonant frequency (*f*res). **Table S17** Reference equations for tidal breathing, exhaled nitric oxide, multiple breath washout and forced oscillation technique measures in 5–11-week South African infants.
