# Supplementary material for: Lung function and exhaled nitric oxide in healthy unsedated African infants
Source: Respirology. 2015 Jul 1;20(7):1108–14. doi: 10.1111/resp.12579 (PMC4623783; doi:10.1111/resp.12579)
Supplement: Supplementary file 1 — Appendix S1 Statistical analysis. Table S1 Demographics and socioeconomic characteristics of participants by study site. Table S2 Univariate and multivariate analysis for tidal volume. Table S3 Univariate and multivariate analysis for respiratory rate. Table S4 Univariate and multivariate analysis for minute ventilation. Table S5 Univariate and multivariate analysis for mean tidal inspiratory flow. Table S6 Univariate and multivariate analysis for mean tidal expiratory flow. Table S7 Univariate and multivariate analysis for time to peak tidal expiratory flow over total expiratory time (tPTEF/tE). Table S8 Univariate analysis, multivariate analysis and reference equation for exhaled nitric oxide (eNO; A); (B) log(eNO). Table S9 Univariate and multivariate analysis for nitric oxide output. Table S10 Univariate and multivariate analysis for functional residual capacity (FRC). Table S11 Univariate analysis for the first moment ratio (M0/M1). Table S12 Univariate analysis for second moment ratio (M0/M2). Table S13 Univariate analysis for the lung clearance index (LCI). Table S14 Univariate and multivariate analysis for respiratory system resistance (R). Table S15 Univariate and multivariate analysis for respiratory system compliance (C). Table S16 Univariate and multivariate analysis for resonant frequency (fres). Table S17 Reference equations for tidal breathing, exhaled nitric oxide, multiple breath washout and forced oscillation technique measures in 5–11-week South African infants. [file resp0020-1108-sd1.docx]

supplementARY INFORMATION

**Lung function and exhaled nitric oxide in healthy unsedated African infants**

Diane Gray^1^, Lauren Willemse^1^, Ane Visagie^1^, Emilee Smith^2^, Dorottya Czövek^3,4^ Peter D. Sly^4^, Zoltán Hantos^3,4^ Graham L Hall^5^, Heather J Zar^1^

^1^Department of Paediatrics and Child Health, Red Cross War Memorial Children’s Hospital, and MRC Unit on Child & Adolescent Lung Health, University of Cape Town, South Africa

^2^Division of Epidemiology and Biostatistics, School of Public Health & Family Medicine, University of Cape Town, South Africa

^3^Department of Medical Physics and Informatics, University Of Szeged, Hungary

^4^Queensland Children's Medical Research Institute, University of Queensland, Australia

^5^Telethon Kids Institute, University of Western Australia, Australia

**APPENDIX 1-Statistical analysis**

Statistical analyses were performed using STATA 13 for windows (STATA Corporation, College Station, Texas, USA). Weight and length for age z scores were calculated using the WHO Child Growth Standards “I grow up” STATA package.^1^ Birth weight and birth length z scores were calculated using the Fenton 2013 growth charts for boys and girls based on gestational age, birth weight and length.^2^ Reference equations were fitted using a stepwise linear regression model with the significance level for removal set to significance levels above 0.05. The linear predictions from the reference equations were used to determine the predicted values for each lung function measurement. Due to having a very skewed distribution, exhaled nitric oxide was also analyzed as a log transformed variable. However, the significant predictors resulting from the multivariate analysis (table E7a) of the transformed variable was not different to the analysis of the non-transformed variable (table E7), and hence the non-transformed variable analysis was used to develop a prediction equation, as the effect sizes are easier to interpret. These were compared to the European reference values ^3^ by fitting the published reference equations to the observed data. Significance testing between predicted values was done using two sample Student’s t-test with a 0.05 level of significance.

**References**

1 *WHO Child Growth Standards*. Geneva: World Health Organisation, 2006.

2 Fenton TR, Kim JH. A systematic review and meta-analysis to revise the Fenton growth chart for preterm infants. BMC pediatrics. 2013; **13**: 59.

3 Fuchs O, Latzin P, Thamrin C, Stern G, Frischknecht P, Singer F, Kieninger E, Proietti E, Riedel T, Frey U. Normative data for lung function and exhaled nitric oxide in unsedated healthy infants. Eur Respir J. 2011; **37**: 1208-16.

**Table S1: Demographics and socioeconomic characteristics of participants by study site.**

|  | Mbekweni  (N=176)  N (%) | Newman  (N=187)  N (%) | Total  (N=363)  N (%) |
| --- | --- | --- | --- |
| Male Sex | 81 (46) | 100 (54) | 181 (50) |
| Maternal HIV infection | 61 (35) | 8 (4) | 69 (19) |
| Maternal smoking in pregnancy | 26 (15) | 95 (52) | 121 (34 |
| Caesarean section | 37 (21) | 32 (17) | 69 (19) |
| Exclusively breastfed | 69 (39) | 79 (42) | 148 (41) |
| Maternal SES |  |  |  |
| Lowest SES | 61 (35) | 36 (19) | 97 (27) |
| Low-Moderate SES | 48 (27) | 37 (20) | 85 (23) |
| Moderate-high SES | 41 (23) | 53 (28) | 94 (26) |
| High SES | 26 (15) | 61 (33) | 87 (24) |
| Ethnicity |  |  |  |
| African | 174 (99) | 2 (1) | 176 (48) |
| Mixed ethnicity | 2 (1) | 185 (99) | 187 (51) |

**Table S2: Univariate and multivariate analysis for tidal volume**

|  |  | **Univariate Model** | |  |  |  | **Multivariate model** | |  |  |  |  |
| --- | --- | --- | --- | --- | --- | --- | --- | --- | --- | --- | --- | --- |
|  |  | **Coefficient** | **95% CI** | | **p-value** |  | **Coefficient** | **95% CI** | | **p-value** | **Adj R²** | **RSD*** |
| **Tidal Volume (mL)** | |  |  |  |  |  |  |  |  |  | 37% | 5.07 |
|  | **Age at study date (weeks)** | 1.830 | 1.211 | 2.449 | 0.000 |  | 1.619 | 1.095 | 2.142 | 0.000 |  |  |
|  | **Male sex** | 1.678 | 0.368 | 2.988 | 0.012 |  | 2.166 | 1.092 | 3.329 | 0.000 |  |  |
|  | **Weight-for-age z score** | 3.203 | 2.651 | 3.754 | 0.000 |  | 3.185 | 2.665 | 3.705 | 0.000 |  |  |
|  | Length-for-age z score | 1.582 | 1.137 | 2.027 | 0.000 |  |  |  |  |  |  |  |
|  | Birth weight z score | 2.160 | 1.528 | 2.791 | 0.000 |  |  |  |  |  |  |  |

*** RSD: residual standard deviation**

**Table S3: Univariate and multivariate analysis for respiratory rate**

|  |  | **Univariate Model** | |  |  |  | **Multivariate model** | |  |  |  |  |
| --- | --- | --- | --- | --- | --- | --- | --- | --- | --- | --- | --- | --- |
|  |  | **Coefficient** | **95% CI** | | **p-value** |  | **Coefficient** | **95% CI** | | **p-value** | **Adj R²** | **RSD*** |
| **Respiratory Rate (n.min^-1^)** | |  |  |  |  |  |  |  |  |  | 7% | 11.511 |
|  | **Age at study date (weeks)** | -2.275 | -3.467 | -1.083 | 0.000 |  | -2.103 | -3.291 | -0.915 | 0.001 |  |  |
|  | Male sex | 0.000 | -2.481 | 2.480 | 1.000 |  |  |  |  |  |  |  |
|  | **Weight-for-age z score** | -2.392 | -3.580 | -1.204 | 0.000 |  | -2.294 | -3.471 | -1.119 | 0.000 |  |  |
|  | Length-for-age z score | -1.584 | -2.459 | -0.710 | 0.000 |  |  |  |  |  |  |  |
|  | Birth weight z score | -2.559 | -3.787 | -1.330 | 0.000 |  |  |  |  |  |  |  |

*** RSD: residual standard deviation**

**Table S4: Univariate and multivariate analysis for minute ventilation**

|  |  | **Univariate Model** | |  |  |  | **Multivariate model** | |  |  |  |  |
| --- | --- | --- | --- | --- | --- | --- | --- | --- | --- | --- | --- | --- |
|  |  | **Coefficient** | **95% CI** | | **p-value** |  | **Coefficient** | **95% CI** | | **p-value** | **Adj R²** | **RSD*** |
| **Minute Ventilation (mL.min^-1^)** | |  |  |  |  |  |  |  |  |  | 9% | 294.25 |
|  | Age at study date (weeks) | 9.642 | -21.864 | 41.148 | 0.548 |  |  |  |  |  |  |  |
|  | **Male sex** | 81.801 | 18.064 | 145.538 | 0.012 |  | 80.993 | 18.410 | 143.576 | 0.011 |  |  |
|  | **Weight-for-age z score** | 77.319 | 47.072 | 107.566 | 0.000 |  | 102.115 | 65.752 | 138.479 | 0.000 |  |  |
|  | length-for-age z score | 25.054 | 2.214 | 47.895 | 0.032 |  |  |  |  |  |  |  |
|  | **Birth weight z score** | 15.116 | -17.449 | 47.682 | 0.362 |  | -41.165 | -79.072 | -3.258 | 0.033 |  |  |

*** RSD: residual standard deviation**

**Table S5: Univariate and multivariate analysis for mean tidal inspiratory flow**

|  |  | **Univariate Model** | |  |  |  | **Multivariate model** | |  |  |  |  |
| --- | --- | --- | --- | --- | --- | --- | --- | --- | --- | --- | --- | --- |
|  |  | **Coefficient** | **95% CI** | | **p-value** |  | **Coefficient** | **95% CI** | | **p-value** | **Adj R²** | **RSD** |
| **Mean tidal insp. flow (mL.s^-1^)** | |  |  |  |  |  |  |  |  |  | 15% | 9.780 |
|  | Age at study date (weeks) | 0.585 | -0.497 | 1.667 | 0.288 |  |  |  |  |  |  |  |
|  | **Male sex** | 3.904 | 1.732 | 6.076 | 0.000 |  | 4.334 | 2.264 | 6.404 | 0.000 |  |  |
|  | **Weight-for-age z score** | 3.355 | 2.342 | 4.368 | 0.000 |  | 3.522 | 2.524 | 4.522 | 0.000 |  |  |
|  | length-for-age z score | 1.385 | 0.609 | 2.160 | 0.001 |  |  |  |  |  |  |  |
|  | Birth weight z score | 1.038 | -0.076 | 2.151 | 0.068 |  |  |  |  |  |  |  |

*** RSD: residual standard deviation**

**Table S6: Univariate and multivariate analysis for mean tidal expiratory flow**

|  |  | **Univariate Model** | |  |  |  | **Multivariate model** | |  |  |  |  |
| --- | --- | --- | --- | --- | --- | --- | --- | --- | --- | --- | --- | --- |
|  |  | **Coefficient** | **95% CI** | | **p-value** |  | **Coefficient** | **95% CI** | | **p-value** | **Adj R²** | **RSD** |
| **Mean tidal exp. flow (mL.s^-1^)** | |  |  |  |  |  |  |  |  |  | 4% | 11.775 |
|  | Age at study date (weeks) | 0.103 | -1.123 | 1.329 | 0.869 |  |  |  |  |  |  |  |
|  | Male sex | 2.115 | -0.375 | 4.606 | 0.096 |  |  |  |  |  |  |  |
|  | **Weight-for-age z score** | 2.103 | 0.903 | 3.303 | 0.001 |  | 2.979 | 1.525 | 4.435 | 0.000 |  |  |
|  | length-for-age z score | 0.485 | -0.408 | 1.378 | 0.286 |  |  |  |  |  |  |  |
|  | **Birth weight z score** | 0.121 | -1.148 | 1.390 | 0.851 |  | -1.667 | -3.177 | -0.158 | 0.030 |  |  |

*** RSD: residual standard deviation**

**Table S7: Univariate and multivariate analysis for time to peak tidal expiratory flow over total expiratory time (t_PTEF_/t_E_)**

|  |  | **Univariate Model** | |  |  |  | **Multivariate model** | |  |  |  |  |
| --- | --- | --- | --- | --- | --- | --- | --- | --- | --- | --- | --- | --- |
|  |  | **Coefficient** | **95% CI** | | **p-value** |  | **Coefficient** | **95% CI** | | **p-value** | **Adj R²** | **RSD*** |
| **t_PTEF_/t_E_ %** | |  |  |  |  |  |  |  |  |  | 1% | 11.974 |
|  | Age at study date (weeks) | -0.273 | -1.497 | 0.951 | 0.661 |  |  |  |  |  |  |  |
|  | **Male sex** | -3.133 | -5.609 | -0.657 | 0.013 |  | -3.078 | -5.607 | -0.549 | 0.017 |  |  |
|  | Weight-for-age z score | 0.369 | -0.854 | 1.593 | 0.553 |  |  |  |  |  |  |  |
|  | length-for-age z score | -0.251 | -1.151 | 0.649 | 0.584 |  |  |  |  |  |  |  |
|  | Birth weight z score | 0.555 | -0.707 | 1.818 | 0.387 |  |  |  |  |  |  |  |

*** RSD: residual standard deviation**

**Table S8**

**A: Univariate analysis, multivariate analysis and reference equation for exhaled nitric oxide (eNO)**

|  |  | **Univariate Model** | |  |  |  | **Multivariate model** | |  |  |  |  |
| --- | --- | --- | --- | --- | --- | --- | --- | --- | --- | --- | --- | --- |
|  |  | **Coefficient** | **95% CI** | | **p-value** |  | **Coefficient** | **95% CI** | | **p-value** | **Adj R²** | **RSD*** |
| **eNO ppb** | |  |  |  |  |  |  |  |  |  | 9.5% | 6.544 |
|  | **Age at study date (weeks)** | 0.931 | 0.234 | 1.628 | 0.009 |  | 1.001 | 0.324 | 1.68 | 0.004 |  |  |
|  | Male sex | -0.339 | -1.776 | 1.097 | 0.643 |  |  |  |  |  |  |  |
|  | **Weight-for-age z score** | 0.581 | -0.141 | 1.304 | 0.114 |  | 0.988 | 0.272 | 1.703 | 0.007 |  |  |
|  | Length-for-age z score | 0.315 | -0.207 | 0.837 | 0.236 |  |  |  |  |  |  |  |
|  | Birth weight z score | 0.886 | 0.152 | 1.620 | 0.018 |  |  |  |  |  |  |  |
|  | **Minute ventilation** | -0.005 | -0.008 | -0.003 | 0.000 |  | -0.006 | -0.009 | -0.004 | 0.000 |  |  |

*** RSD: residual standard deviation**

**B: Univariate analysis, multivariate analysis and reference equation for exhaled nitric oxide, log(eNO)**

|  |  | **Univariate Model** | |  |  |  | **Multivariate model** | |  |  |  |  |
| --- | --- | --- | --- | --- | --- | --- | --- | --- | --- | --- | --- | --- |
|  |  | **Coefficient** | **95% CI** | | **p-value** |  | **Coefficient** | **95% CI** | | **p-value** | **Adj R²** | **RSD** |
| **Log(eNO)** | |  |  |  |  |  |  |  |  |  | 5% | 0.9175 |
|  | Age at study date (weeks) | 0.035 | -0.061 | 0.132 | 0.472 |  |  |  |  |  |  |  |
|  | Male sex | -0.100 | -0.297 | 0.098 | 0.321 |  |  |  |  |  |  |  |
|  | **Weight-for-age z score** | 0.109 | 0.011 | 0.207 | 0.029 |  | 0.153 | 0.053 | 0.253 | 0.003 |  |  |
|  | Length-for-age z score | 0.063 | -0.008 | 0.134 | 0.083 |  |  |  |  |  |  |  |
|  | Birth weight z score | 0.129 | 0.029 | 0.229 | 0.012 |  |  |  |  |  |  |  |
|  | **Minute ventilation** | -0.0006 | -0.0009 | -0.0002 | 0.000 |  | -0.0006 | -0.0009 | -0.0003 | 0.000 |  |  |

*** RSD: residual standard deviation**

**Table S9: Univariate and multivariate analysis for nitric oxide output**

|  |  | **Univariate Model** | |  |  |  | **Multivariate model** | |  |  |  |  |
| --- | --- | --- | --- | --- | --- | --- | --- | --- | --- | --- | --- | --- |
|  |  | **Coefficient** | **95% CI** | | **p-value** |  | **Coefficient** | **95% CI** | | **p-value** | **Adj R²** | **RSD** |
| **NO output (nL.min^-1^)** | |  |  |  |  |  |  |  |  |  | 3% | 21.033 |
|  | **Age at study date (weeks)** | 2.827 | 0.657 | 4.996 | 0.011 |  | 2.963 | 0.785 | 5.143 | 0.008 |  |  |
|  | Male sex | -0.784 | -5.255 | 3.686 | 0.730 |  |  |  |  |  |  |  |
|  | **Weight-for-age z score** | 2.880 | 0.649 | 5.112 | 0.012 |  | 2.668 | 0.438 | 4.899 | 0.019 |  |  |
|  | Length-for-age z score | 1.077 | -0.545 | 2.699 | 0.192 |  |  |  |  |  |  |  |
|  | Birth weight z score | 2.574 | 0.287 | 4.861 | 0.027 |  |  |  |  |  |  |  |

*** RSD: residual standard deviation**

**Table S10: Univariate and multivariate analysis for functional residual capacity (FRC)**

|  |  | **Univariate Model** | |  |  |  | **Multivariate model** | |  |  |  |  |
| --- | --- | --- | --- | --- | --- | --- | --- | --- | --- | --- | --- | --- |
|  |  | **Coefficient** | **95% CI** | | **p-value** |  | **Coefficient** | **95% CI** | | **p-value** | **Adj R²** | **RSD*** |
| **FRC** | **(mL)** |  |  |  |  |  |  |  |  |  | 11% | 16.314 |
|  | **Age at study date (weeks)** | 2.684 | 0.934 | 4.434 | 0.003 |  | 2.148 | 0.428 | 3.868 | 0.015 |  |  |
|  | Male sex | -3.002 | -6.624 | 0.621 | 0.104 |  |  |  |  |  |  |  |
|  | **Weight-for-age z score** | 5.035 | 3.314 | 6.756 | 0.000 |  | 3.638 | 1.579 | 5.697 | 0.001 |  |  |
|  | Length-for-age z score | 2.445 | 1.148 | 3.741 | 0.000 |  |  |  |  |  |  |  |
|  | **Birth weight z score** | 4.624 | 2.846 | 6.402 | 0.000 |  | 2.219 | 0.086 | 4.352 | 0.042 |  |  |

*** RSD: residual standard deviation**

**Table S11: Univariate analysis for the first moment ratio (M0/M1)**

|  |  | **Univariate Model** | |  |  |  |
| --- | --- | --- | --- | --- | --- | --- |
|  |  | **Coefficient** | **95% CI** | | **p-value** |  |
| **M0/M1** | |  |  |  |  |  |
|  | Age at study date (weeks) | 0.008 | -0.005 | 0.022 | 0.227 |  |
|  | Male sex | 0.022 | -0.006 | 0.050 | 0.124 |  |
|  | Weight-for-age z score | 0.006 | -0.008 | 0.019 | 0.397 |  |
|  | Length-for-age z score | 0.003 | -0.007 | 0.013 | 0.510 |  |
|  | Birth weight z score | 0.008 | -0.006 | 0.023 | 0.237 |  |

**Table S12: Univariate analysis for second moment ratio (M0/M2)**

|  |  | **Univariate Model** | |  |  |  |
| --- | --- | --- | --- | --- | --- | --- |
|  |  | **Coefficient** | **95% CI** | | **p-value** |  |
| **M0/M2** | |  |  |  |  |  |
|  | Age at study date (weeks) | 0.068 | -0.478 | 0.183 | 0.250 |  |
|  | Male sex | 0.187 | -0.049 | 0.424 | 0.120 |  |
|  | Weight-for-age z score | 0.047 | -0.068 | 0.163 | 0.420 |  |
|  | length-for-age z score | 0.035 | -0.049 | 0.120 | 0.408 |  |
|  | Birth weight z score | 0.074 | -0.046 | 0.196 | 0.224 |  |

**Table S13: Univariate analysis for the lung clearance index (LCI)**

|  |  | **Univariate Model** | |  |  |  |
| --- | --- | --- | --- | --- | --- | --- |
|  |  | **Coefficient** | **95% CI** | | **p-value** |  |
| **LCI** | **(n FRC turnovers)** |  |  |  |  |  |
|  | Age at study date (weeks) | -0.004 | -0.049 | 0.041 | 0.863 |  |
|  | Male sex | 0.038 | -0.055 | 0.130 | 0.423 |  |
|  | Weight-for-age z score | -0.033 | -0.078 | 0.012 | 0.150 |  |
|  | length-for-age z score | -0.011 | -0.044 | 0.022 | 0.520 |  |
|  | Birth weight z score | -0.004 | -0.051 | 0.043 | 0.857 |  |

**Table S14: Univariate and multivariate analysis for respiratory system resistance (R)**

|  |  | **Univariate Model** | |  |  |  | **Multivariate model** | |  |  |  |  |
| --- | --- | --- | --- | --- | --- | --- | --- | --- | --- | --- | --- | --- |
|  |  | **Coefficient** | **95% CI** | | **p-value** |  | **Coefficient** | **95% CI** | | **p-value** | **Adj R²** | **RSD*** |
| **R (cmH_2_O.s.L^-1^)** | |  |  |  |  |  |  |  |  |  | 4% | 15.51 |
|  | Age at study date (weeks) | -0.649 | -2.465 | 1.167 | 0.483 |  |  |  |  |  |  |  |
|  | **Male sex** | 5.131 | 1.553 | 8.709 | 0.005 |  | 4.918 | 1.292 | 8.544 | 0.008 |  |  |
|  | **Weight-for-age z score** | 1.164 | -0.613 | 2.941 | 0.198 |  | 2.994 | 0.731 | 5.257 | 0.010 |  |  |
|  | **length-for-age z score** | -0.760 | -2.102 | 0.581 | 0.266 |  | -1.936 | -3.649 | -0.223 | 0.027 |  |  |
|  | Birth weight z score | 0.508 | -1.309 | 2.325 | 0.582 |  |  |  |  |  |  |  |

*** RSD: residual standard deviation**

**Table S15: Univariate and multivariate analysis for respiratory system compliance (C)**

|  |  | **Univariate Model** | |  |  |  | **Multivariate model** | |  |  |  |  |
| --- | --- | --- | --- | --- | --- | --- | --- | --- | --- | --- | --- | --- |
|  |  | **Coefficient** | **95% CI** | | **p-value** |  | **Coefficient** | **95% CI** | | **p-value** | **Adj R²** | **RSD*** |
| **C (mL.cmH_2_O^-1^)** | |  |  |  |  |  |  |  |  |  | 5% | 0.42585 |
|  | Age at study date (weeks) | 0.048 | -0.002 | 0.098 | 0.062 |  |  |  |  |  |  |  |
|  | **Male sex** | **-0.177** | **-0.276** | **-0.078** | **0.000** |  | -0.160 | -0.257 | -0.058 | 0.002 |  |  |
|  | Weight-for-age z score | 0.039 | -0.010 | 0.088 | 0.120 |  |  |  |  |  |  |  |
|  | **Length-for-age z score** | **0.054** | **0.018** | **0.091** | **0.004** |  | 0.049 | 0.010 | 0.012 | 0.085 |  |  |
|  | Birth weight z score | 0.057 | 0.007 | 0.107 | 0.025 |  |  |  |  |  |  |  |

*** RSD: residual standard deviation**

**Table S16: Univariate and multivariate analysis for resonant frequency (*f*res)**

|  |  | **Univariate Model** | |  |  |  | **Multivariate model** | |  |  |  |  |
| --- | --- | --- | --- | --- | --- | --- | --- | --- | --- | --- | --- | --- |
|  |  | **Coefficient** | **95% CI** | | **p-value** |  | **Coefficient** | **95% CI** | | **p-value** | **Adj R²** | **RSD*** |
| ***f*res (Hz)** | |  |  |  |  |  |  |  |  |  | 4% | 5.9966 |
|  | Age at study date (weeks) | -0.583 | -1.284 | 0.117 | 0.102 |  |  |  |  |  |  |  |
|  | **Male sex** | 1.944 | 0.557 | 3.331 | 0.006 |  | 1.659 | 0.257 | 3.061 | 0.020 |  |  |
|  | Weight-for-age z score | -0.981 | -1.663 | -0.299 | 0.005 |  |  |  |  |  |  |  |
|  | **Length-for-age z score** | -0.795 | -1.308 | -0.282 | 0.003 |  | -0.738 | -1.258 | -0.222 | 0.005 |  |  |
|  | Birth weight z score | -0.867 | -1.563 | -0.171 | 0.015 |  |  |  |  |  |  |  |

*** RSD: residual standard deviation**

**Table S17: Reference equations for tidal breathing, exhaled nitric oxide, multiple breath washout and forced oscillation technique measures in 5-11 week South African infants**

|  | **Regression equations** | **Adj R^2^** | **RSD** |
| --- | --- | --- | --- |
| **Tidal breathing** |  |  |  |
| Tidal volume | 22.932 + 1.619 * (age weeks) + 2.166 * (male) + 3.185 * (weight for age) | 37% | 5.1 |
| Respiratory rate | 62.859 – 2.103 * (age weeks) – 2.294 * (weight for age) | 7% | 11.5 |
| Minute ventilation | 1,590.33 + 80.993 * (male) + 102.115 * (weight for age) – 41.165* (birth weight z score) | 9% | 294.3 |
| Mean tidal inspiratory flow rate | 59.588 + 4.334 * (Male) + 3.522 * (weight for age) | 15% | 9.8 |
| Mean tidal expiratory flow rate | 49.799 + 2.979 * (weight for age) - 1.667 * (birth weight z score) | 4% | 11.8 |
| **Exhaled NO** |  |  |  |
| Exhaled nitric oxide | 13.32 + 1.002 * (age weeks) + 0.988 * (weight for age z score) – 0.006 * (minute ventilation) | 9.5% | 6.5 |
| NO output | 12.11 + 2.963 * (age weeks) + 2.668 * (weight age z) | 3% | 21.0 |
| **Multiple breath washout** |  |  |  |
| FRC | 64.970 + 2.148* (Age at study date) + 3.638* (weight for age z score) + 2.219* (birth weight z score) | 11% | 16.3 |
| M0/M1, M0/M2 and LCI: see mean data table | | | |
| **Forced oscillation technique** |  |  |  |
| Resistance | 45.835 + 4.918 * (male) + 2.994 * (weight for age) – 1.936 * (length for age) | 4% | 15.5 |
| Compliance | 1.06 – 0.160 * (male) + 0.049 * (length for age) | 5% | 0.4 |
| Resonant frequency | 19.933 + 1.779 * (male) – 0.721 * (weight for age) + 1.325 * (Maternal passive smoker) + 3.035*(Maternal active smoker) | 7% | 5.9 |

RSD: residual standard deviation
